# Green Synthesis of Silver Nanoparticles Using *Pinus eldarica* Bark Extract

**DOI:** 10.1155/2013/639725

**Published:** 2013-09-08

**Authors:** Siavash Iravani, Behzad Zolfaghari

**Affiliations:** Department of Pharmacognosy and Isfahan Pharmaceutical Sciences Research Center, School of Pharmacy and Pharmaceutical Sciences, Isfahan University of Medical Sciences, Isfahan 81744-176, Iran

## Abstract

Recently, development of reliable experimental protocols for synthesis of metal nanoparticles with desired morphologies and sizes has become a major focus of researchers. Green synthesis of metal nanoparticles using organisms has emerged as a nontoxic and ecofriendly method for synthesis of metal nanoparticles. The objectives of this study were production of silver nanoparticles using *Pinus eldarica* bark extract and optimization of the biosynthesis process. The effects of quantity of extract, substrate concentration, temperature, and pH on the formation of silver nanoparticles are studied. TEM images showed that biosynthesized silver nanoparticles (approximately in the range of 10–40 nm) were predominantly spherical in shape. The preparation of nano-structured silver particles using *P. eldarica* bark extract provides an environmentally friendly option, as compared to currently available chemical and/or physical methods.

## 1. Introduction 

In recent years, green synthesis of metal nanoparticles is an interesting issue of the nanoscience and nanobiotechnology. There is a growing attention to biosynthesis the metal nanoparticles using organisms. Among these organisms, plants seem to be the best candidate and they are suitable for large-scale biosynthesis of nanoparticles. Nanoparticles produced by plants are more stable, and the rate of synthesis is faster than that in the case of other organisms. Moreover, the nanoparticles are more various in shape and size in comparison with those produced by other organisms [1, 2]. 

Silver nanoparticles have drawn the attention of researchers because of their suitable applications in the fields of electronic, material science, and medicine [3, 4]. For instance, antimicrobial properties of silver nanoparticles caused the use of these nanometals in different fields of medicine, various industries, animal husbandry, packaging, accessories, cosmetics, health, and military. Silver nanoparticles show potential antimicrobial effects against infectious organisms such as *Escherichia coli*, *Bacillus subtilis*, *Vibrio cholerae*, *Pseudomonas aeruginosa*, *Syphilis typhus*, and *Staphylococcus aureus* [5, 6]. Moreover, these nanoparticles have drawn the attention of researchers because of their extensive applications in areas such as mechanics, optics, biomedical sciences, chemical industry, electronics, space industries, drug-gene delivery, energy science, catalysis [7, 8], optoelectronic devices [9, 10], photo-electrochemical applications [11], and nonlinear optical devices [12, 13].

Antioxidant action of phenolic compounds is due to their high tendency to chelate metals. These compounds consist of catechin, taxifolin, procyanidins of various chain lengths formed by catechin and epicatechin units, and phenolic acids. Phenolic compounds possess hydroxyl and carboxyl groups. These compounds may inactivate iron ions by chelating and additionally suppressing the superoxide-driven Fenton reaction, which is believed to be the most important source of reactive oxygen species (ROS). Therefore, plants with high content of phenolic compounds (e.g., Pinus species) are one of the best candidates for nanoparticle synthesis. Pine bark extract contains polyphenolic compounds which have considerable antioxidant activities. The objectives of this study were production of silver nanoparticles using *Pinus eldarica* bark extract and optimization of the biosynthesis process.

## 2. Materials and Methods

### 2.1. Plant Material

*Pinus eldarica* bark specimens were collected from a population growing in Isfahan (32°38′N 51°39′E, altitude, 1590 m) from Isfahan. The plant was identified by the Botany Department of the Faculty of Sciences at the University of Isfahan. The samples were collected between August and September 2010. The specimens were dried at room temperature, ground by using a conventional grinder, and stored at 4°C. 

### 2.2. Plant Characteristics

*Pinus eldarica* (Pinaceae) is a medium-sized tree, reaching 12–15 m high. The bark is brownish gray or light gray, not flaking, and head broad-topped. The leaves are stiff, 6–9 cm long, and green. The cones are pedunculate, solitary or in pairs, and light reddish brown. Scales irregularly rhombic, glossy, smooth, the whitish-gray apophysis concave: seeds blackish, 6-7 mm long, the reddish-brown wing 18–28 mm long [14]. 

### 2.3. Biosynthesis and Characterization of Silver Nanoparticles

*P. eldarica* bark extract was used as a reducing agent for the development of silver nanoparticles. Fifty g of pine bark powders was added in 250 mL deionized water in 500 mL Erlenmeyer flask boiled for approximately 15 min. Whatman filter paper was used for the filtration of boiled materials to prepare the aqueous pine bark extract, which was used for metal nanoparticle synthesis. The reaction mixtures contained the following ingredients (final concentrations): AgNO_3_ (1, 2, 4, and 6 mM) as the substrate, different quantities of *P. eldarica* bark extract, and phosphate buffer (pH = 3, 5, 7, 9, and 11). UV absorption of colloidal suspension (hydrosol) of silver nanoparticles was used as an easy and quick assay to check production of nanoparticles. Absorption spectra were measured on a Shimadzu (UVmini-1240, Japan) spectrophotometer. Transmission electron microscopy (TEM) analysis was performed on selected samples in order to investigate the process of formation of silver nanoparticles and study the size and shape of them. Micrographs were obtained using CM 200 FEG Phillips transmission electron microscope. 

## 3. Results and Discussion 

### 3.1. Visual Inspection

When *P. eldarica* bark extract was exposed to Ag^+^ ions (AgNO_3_, 1 mM), the color of the reaction mixture turned to yellowish brown and then to dark brown, which was in agreement with the previous studies, and was considered as the formation of silver nanoparticles [15, 16]. The appearance of dark brown seems to be due to excitation of surface plasmon resonance in the nanoparticles. 

### 3.2. Monitoring the Production of Silver Nanoparticles

**Figure 1:**
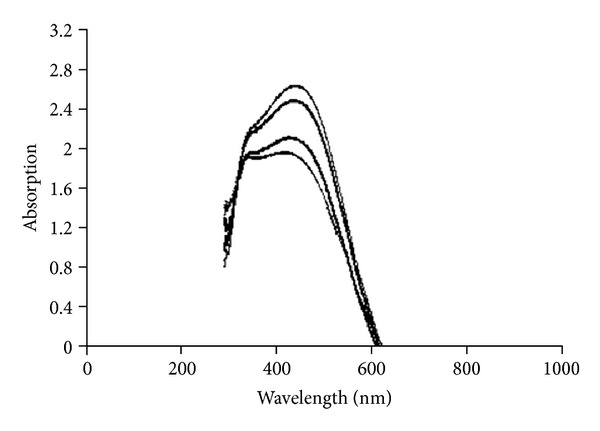
UV/Vis absorption spectrum of the produced colloidal silver. The spectrum was obtained at different time points after the start of AgNO_3_ (1 mM) reduction.

In order to study and optimize the production of nanoparticles, we needed an easy and cheap method to monitor the nanoparticles production. Most of the researchers have used the optical absorption of colloidal silver as an indicator of production of silver nanoparticles [1, 2, 17–22]. We examined the UV/Vis absorption spectrum of colloidal Ag to verify this. The *λ*_max⁡_ was approximately 430 nm (Figure 1). By plotting UV/Vis absorption of the reaction mixture against time, time course of the reaction was obtained. During the reaction period, an increase in absorbance was observed in this wavelength, which can be due to the increase in production of colloidal silver nanoparticles [23, 24]. 

The important challenges frequently encountered in the biosynthesis of nanoparticles are to control the shape and size of the particles as well as to achieve the monodispersity in solution phase. Several factors such as substrate concentration, electron donor, reaction or incubation time, pH, temperature, buffer strength, mixing speed, and light need to be optimized.

### 3.3. Effect of Substrate Concentration

**Figure 2:**
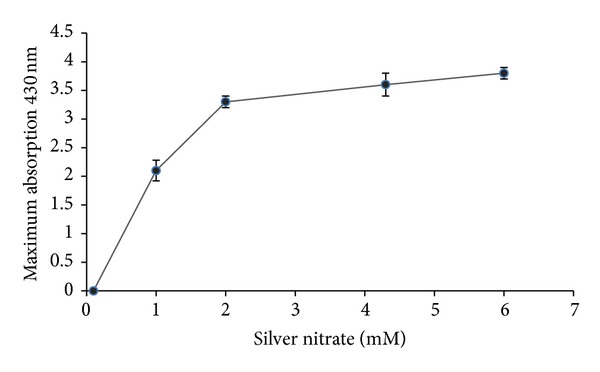
Effect of different concentrations of the substrate: absorption spectra of maximum production of silver nanoparticles against various concentrations of AgNO_3_ (1, 2, 4, and 6 mM) were read and recorded (*n* value = 3).

One of the important measures to make the reaction more economical and efficient is finding the maximum concentration of substrate which could be converted to final product. The results obtained from time course of reaction indicated that by gradual increase in concentration of AgNO_3_, the nanoparticle production was also increased (Figure 2). 

### 3.4. Effect of Different Quantities of *P. eldarica* Bark Extract

**Figure 3:**
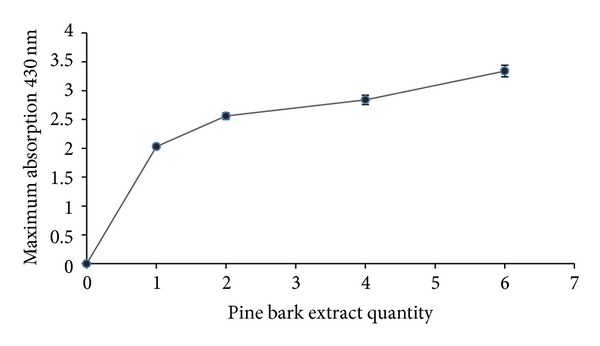
Effect of different quantities of *P. eldarica* bark extract: absorption spectra of maximum production of silver nanoparticles against various amounts of *P. eldarica* bark extract (×1, ×2, ×4, and ×6) were read and recorded (*n* value = 3).

 The possibility of controlling the properties of nanoparticles by changing the composition of the reaction mixture has resulted in the use of different amount of biomass or cell extract in order to form nanoparticles with desired morphology and size. Different pine bark extract quantities were used for the synthesis of silver nanoparticles. The pine bark extract varied from 1, 2, 4, and 6 mL in 50 mL of 1 mM silver nitrate solution. As a result, larger quantities of bark extract lead to an increase in peak absorbance in UV/Vis spectrum. By increasing the extract concentrations, nanoparticle production was also increased, but this relationship was not linear (Figure 3). Moreover, decrease in particle size of Ag nanoparticles has been observed due to an increase in extract amount.

### 3.5. Effect of pH

**Figure 4:**
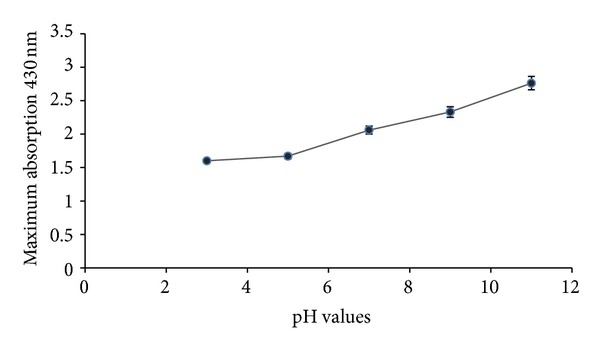
Effect of different pH on nanoparticle production: absorption spectra of maximum production of silver nanoparticles against 5 pH values of the reaction mixture were read and recorded (*n* value = 3).

 The solution pH is a critical factor in controlling the size and morphology of nanoparticles and in the location of nanoparticle deposition [25–27]. The reduction of silver was performed at pH 3, 5, 7, 9, and 11 with *P. eldarica* bark extract. By increasing the pH of the reaction mixture, an increase in absorbance was observed, which can be due to the increase in production of colloidal silver nanoparticles and reduction rate (Figure 4). It seems that pH affects the amount of nanoparticle production and stability of them. Furthermore, pH influenced the rate of the reduction reaction. The reaction mixture turned brown when silver was reduced, and the reaction mixture coloring accelerated when increasing pH. Furthermore, the formation of large sized silver nanoparticles was observed at lower or acidic pH; while higher or alkaline pH highly dispersed, small sized nanoparticles tended to form. The results were in agreement with the previous studies. For instance, Gardea-Torresdey et al. [28] found that pH is an important factor in the biosynthesis of colloidal gold using alfalfa biomass and concluded that the size of nanoparticles varied with the change in pH. Mock et al. [29] also have reached similar conclusions and reported that pH is responsible for the formation of nanoparticles of various shapes and size as different plant extracts and even the extracts coming from different parts of the same plant may have different pH values which further need optimization for the efficient synthesis of nanoparticles. It has been reported by several researchers that larger nanoparticles formed at lower pH [2–4] as compared to higher pH. Moreover, Armendariz et al. [30] reported that the size of gold nanoparticle produced by *Avena sativa *was highly dependent on the pH value. At pH 2, large size nanoparticles (25–85 nm) were formed albeit in a small quantity, but at pH 3 and 4, smaller sized nanoparticles were formed in a large quantity. They speculated that at low pH (pH 2), the gold nanoparticles prefer to aggregate to form larger nanoparticles rather than to nucleate and form new nanoparticles. In contrast, at pH 3 and 4, more functional groups (carbonyl and hydroxyl) are available for gold binding; thus a higher number of Au (III) complexes would bind to the biomass at the same time. Dwivedi and Gopal [31] revealed that silver and gold nanoparticles are stable in a wider range of pH as they observed very small variation in the zeta potential values between pH 2 and 10 in their study using *Chenopodium album*. Veerasamy et al. [32], while working on mangosteen leaf extract, reported that at low pH, aggregation of silver nanoparticles is favoured over the nucleation. However, higher pH facilitates the nucleation and subsequent formation of large number of nanoparticles with smaller diameter.

### 3.6. Effect of Temperature

**Figure 5:**
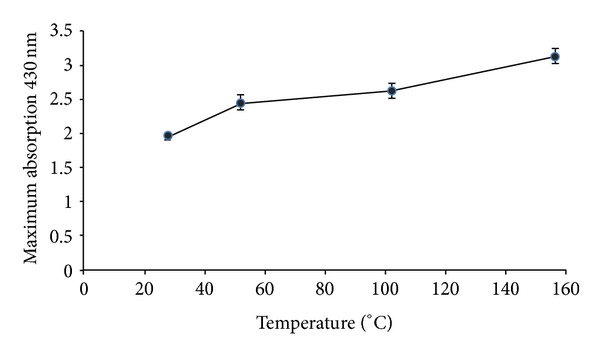
Effect of reaction temperature: absorption spectra of maximum production of silver nanoparticles against different reaction temperature were read and recorded (*n* value = 3).

 Temperature might be one of the crucial factors dominating the size and shape of nanoparticles. In order to investigate the effect of temperature, the reaction mixture was heated at different temperatures (25°C, 50°C, 100°C, and 150°C). Samples were collected from the reaction mixture, and surface plasmon resonancespectra were taken. By increasing reaction temperature, an increase in absorbance was observed, which can be due to the increase in production of colloidal silver nanoparticles and reduction rate (Figure 5). Moreover, when the reaction temperature was increased from 25 to 150°C, the size of silver nanoparticles became smaller which resulted into sharpness of plasmon resonance band of them.

### 3.7. TEM Analysis of Produced Silver Nanoparticles

**Figure 6:**
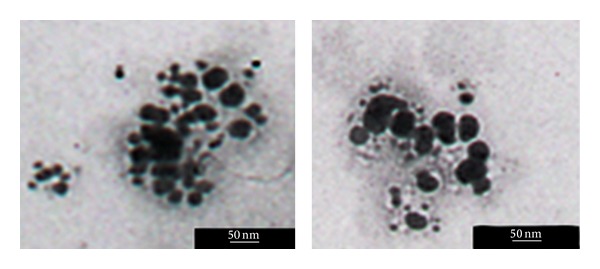
TEM micrographs recorded from the drop-coated film of the silver nanoparticles synthesized by treating the silver nitrate solution with *P. eldarica* bark extract.

Figure 6 shows TEM images recorded from the drop-coated film of the silver nanoparticles synthesized by treating the silver nitrate solution with the pine bark extract. Silver nanoparticles, mainly spherical assemblies, were obtained by room-temperature synthesis. Biosynthesized silver nanoparticles (approximately in the range of 10–40 nm) were predominantly spherical in shape. The reduction and growth of silver nuclei using pine bark extract as a green process present a reliable and economic method, taking advantages of an efficient bioresource. 

## 4. Conclusions

In conclusion, silver nanoparticles were successfully produced using *P. eldarica* bark extract. Characterization by UV-visible and TEM techniques confirmed the reduction of silver ions to silver nanoparticles. The preparation of nanostructured silver particles using *P. eldarica* bark extract provides an environmentally friendly option, as compared to currently available chemical and/or physical methods. 

Various chemical, physical and biological synthetic methods have been developed to obtain metal nanoparticles of various shapes and sizes, including laser ablation, gamma irradiation, electron irradiation, chemical reduction, photochemical methods, microwave processing, and biological synthetic methods. The organisms used in biological synthesis of nanoparticles vary from simple prokaryotic bacterial cells to complex eukaryotes. Plants are able to reduce the metal ions faster than fungi or bacteria. Furthermore, in order to use an easy and safe green method in scaleup and industrial production of well-dispersed metal nanoparticles, plant extract is better than plant biomass or living plant in the rate of production. Problems experienced in biological synthesis of metal nanoparticles are stability and aggregation of nanoparticles, control of crystal growth, morphology, size, and size distribution. Moreover, separation of produced nanoparticles for further applications is still an important issue.
